# Quality of Life and Functional Health Status of Long-Term Meditators

**DOI:** 10.1155/2012/350674

**Published:** 2012-05-07

**Authors:** Ramesh Manocha, Deborah Black, Leigh Wilson

**Affiliations:** ^1^Discipline of Psychiatry, Sydney Medical School, University of Sydney, Royal North Shore Hospital, St. Leonards, NSW 2065, Australia; ^2^Faculty of Health Sciences, University of Sydney, Cumberland Campus, Lidcombe, NSW 2141, Australia

## Abstract

*Background*. There is very little data describing the long-term health impacts of meditation. *Aim*. To compare the quality of life and functional health of long-term meditators to that of the normative population in Australia. *Method*. Using the SF-36 questionnaire and a Meditation Lifestyle Survey, we sampled 343 long-term Australian Sahaja Yoga meditation practitioners and compared their scores to those of the normative Australian population. *Results*. Six SF-36 subscales (bodily pain, general health, mental health, role limitation—emotional, social functioning, and vitality) were significantly better in meditators compared to the national norms whereas two of the subscales (role limitation—physical, physical functioning) were not significantly different. A substantial correlation between frequency of mental silence experience and the vitality, general health, and especially mental health subscales (*P* < 0.005) was found. *Conclusion*. Long-term practitioners of Sahaja yoga meditation experience better functional health, especially mental health, compared to the general population. A relationship between functional health, especially mental health, and the frequency of meditative *experience* (mental silence) exists that may be causal. Evidence for the potential role of this definition of meditation in enhancing quality of life, functional health and wellbeing is growing. Implications for primary mental health prevention are discussed.

## 1. Introduction

Meditation is widely perceived as an effective method of reducing stress and enhancing wellbeing. The US Centers for Disease Control and Prevention's 2002 National Health Interview Survey administered to 31,000 representative adults showed that 8% of respondents had practiced meditation at some time [2]. The Australian Community Survey found that 1.5 million Australians had tried meditation in the past 12 months and that while 29% of Australians found prayer to be a source of peace and wellbeing, 24% used meditation for the same thing. Remarkably, despite only about 20% of Australians attend church monthly or more often, around 33% of Australians pray or meditate at least weekly [3, 4].

Health professionals are also enthusiastic about meditation despite a lack of formal education about it; a survey of Australian GPs found that almost 80% of respondents had recommended meditation to patients at some time in the course of their practice, yet less than 35% had any formal training or education in the field [5]. Mindfulness has become particularly popular in recent years both amongst consumers and health professionals. The growing popularity of meditation as a lifestyle and health enhancing strategy warrants more detailed assessment of its impacts on those who choose to practice it regularly over long periods of time. In spite of this, there remains a lack of evidence about the long-term health effects of regular meditation [6, 7].

Despite the fact that there are hundreds, if not thousands, of interventional studies of meditation in the literature, they are limited to relatively short durations of practice, usually several weeks [8]. Yet meditation, as it was traditionally conceived, was intended to be a life-long practice, the benefits of which were not necessarily expected to manifest in the short term. Unfortunately, interventional studies to assess benefit (or detriment) over periods of years and decades are difficult to accomplish and are prone to a wide range of confounding effects and logistical challenges. Meditation originated as spiritual practice in India, initially linked with Hindu spiritual philosophies such as yoga, later spread more widely in association with Buddhist spirituality to the orient. In different places, it underwent transformations giving rise to traditions such as Zen. Since the early 20th century, meditative practices, along with Eastern spiritual ideas, have become popular in the West, and in the last few decades they have become the focus of scientific attention.

Independently of this, a growing body of mostly epidemiological evidence has emerged pointing to a significant association between what has come to be termed “religiosity” and health [9]. Studies of specific religious groups point to the positive impact of prayer and religious practices on health [1]. Pertinent examples of this relationship are the numerous studies in Seventh Day Adventists and Meisenhelder's 2001 study investigating Presbyterian pastors [1, 10]. Studies demonstrate that Seventh Day Adventists exhibit specific physical health advantages when compared to non-Seventh Day Adventists. These include a reduced risk of coronary heart disease and reduced prevalence of chronic conditions. Most of this health benefit appears secondary to the relatively specific diet and lifestyle of Seventh Day Adventists, which includes avoidance of alcohol, tobacco, and meat [10].

Assessment of 1400 Presbyterian pastors by Meisenhelder and Chandler [1] showed that this group had considerably better health compared to USA population norms. After controlling for age and other demographic variables, a small correlation was found between frequency of prayer and certain important health dimensions, particularly mental health, vitality, and general health. The investigators hypothesised that this correlation may be due to the direct effects of prayer, which induces a “meditation-like state” of reduced physiological arousal and includes a dimension of psychological support derived from seeking solace in “a divine other” [1].

Despite the abundance of short term interventional trials of meditation, there is very little in the literature regarding its long-term impacts or its influence on population health. We identified only two notable studies: Reibel investigated health related quality of life and the practice of meditation in a long-term context. In a year-long observational study of an 8-week mindfulness meditation program involving 136 participants, one year after the initial intervention, participants' chronic illness, physical symptoms, and pain had reduced by 28% and psychological distress decreased by 38% [11].

The only other long-term study of meditation was a retrospective assessment conducted by Otis in 1984 [11]. Otis followed up over 1,000 people who had participated in a student meditation instructional programme at the Stanford Research Institute and, counter-intuitively, found that long-term practitioners of meditation described a range of negative effects of the practice. These included antisocial behaviour, anxiety, confusion, and depression and correlated with the length of time the participants had been practicing meditation. Thus the only two studies aimed at assessing the long-term impacts of meditation present a contradictory picture.

Importantly, despite the large number of interventional studies of meditation in the literature, the vast majority are not properly designed to control for confounding, nonspecific effects. Much of this oversight relates to the lack of clarity around the definition of meditation. One definition that has yielded evidence for specific effect in rigorously designed trials is a paradigm of meditation that features the experience of “mind-emptiness” or “mental silence” as its defining characteristic [12]. Sahaja Yoga is a noncommercial form of meditation and is an example of this form of the mental silence approach. In this context, the meditative experience is a state in which the practitioner is fully alert, aware, and in control of their faculties but does not experience any unwanted thought activity. Practitioners describe the experience of thoughtless awareness or “mental silence” as an enhancement of awareness and self-control, enabling them to attend to the demands of the present moment [12]. Studies of the mental silence approach to meditation have been conducted in a range of health conditions [13]. Rigorous randomised trials using active control groups have demonstrated significant effects on mood and airway hyperresponsiveness in asthma sufferers [14] and on depressive mood and work stress in full-time workers [15]. In other studies, promising effects have been shown in depression/anxiety [16, 17], attention deficit and hyperactivity disorder [18], menopausal hot flashes [19], and epilepsy [20]. Brain physiological studies indicate that the meditative experience is highly correlated with specific electrical activation patterns [21]. Thus short-term observational trials support the premise that this technique has measurable specific effects. Data on its long-term effects however are, like most meditation techniques,unavailable.

It is interesting that while epidemiologists have focused on the associations between western (i.e. Judeo Christian) styles of religiosity and health, health professionals, especially medical and psychological, have been focusing on the health effects of eastern (i.e., meditation) styles of religiosity. Clearly there is much to be gained by bringing these two strands of investigation together.

Consequently we designed this study to both provide data regarding the association between several aspects of quality of life, functional health, and wellbeing in a group of people who practiced long-term mental silence meditation, as well as attempt to unify some the data on the relationship between practices that have their origin in either eastern or western religious tradition. We report here the data relating to the quality of life and functional health measures.

## 2. Aim

The primary aim of this study was to compare the quality of life and functional health of a sample of the population who have practised long-term mental silence meditation to that of the normative Australian population. A secondary aim was to explore the relationship between health scores, meditative practice, and meditative experience (mental silence—MS). We report here the data relating to the SF36.

## 3. Method

### 3.1. Study Design

This cross-sectional survey aimed to collect data concerning quality of life and health functionality. We report here the outcomes relating to the Medical Outcomes Study Short Form (SF-36) and the Meditation Lifestyle Survey (MLS). The MLS was developed specifically for the purposes of this study. The study was approved by the Research Ethics Committee of the University of New South Wales.

### 3.2. Recruitment

Sahaja Yoga Meditation (SYM) Centres around Australia were contacted and asked to participate in a cross-sectional study investigating long-term meditation practice. A researcher, no reference needed, travelled to each of the capital cities of Australia and attended the main meditation meeting in each centre. The researcher also attended five one-day and weekend meditation retreats held between six and ten times per year to promote the study. Participation in the study was voluntary and anonymous. At the commencement of each meeting/retreat, the study purpose was explained in detail, as was the anonymous and confidential nature of participation. Sahaja Yoga Meditation Centres do not keep formal membership lists; however informal lists of phone contacts were used to identify other practitioners who may not regularly attend meetings but still consider themselves diligent practitioners.

### 3.3. Sample Size

The researcher attended seven meetings and five weekend retreats. In total, 336 practitioners of meditation attended one of these meetings or retreats. A total of 311 practitioners completed the questionnaire and survey at these meetings/retreats/weekends. Examination of the informal lists revealed a further 215 records; they were contacted and mailed a set of questionnaires. A total of 32 (6%) responded from this group. In total, 343 responses were available for detailed analysis, representing approximately 63% of the total purported SYM practitioner community in Australia or 93% of those practitioners who might be described as “diligent, long-term practitioners”. While the sampling design was opportunistic, the response rate of the survey was such that it was effectively a census. In this context it is reasonable to assume that nonresponder demographic characteristics are highly likely to be similar to those of the responders.

### 3.4. Questionnaires

#### 3.4.1. Medical Outcomes Study Short Form 36 Questionnaire (SF-36)

The Medical Outcomes Study Short Form 36 (MOS SF-36) is a widely used health and quality of life self-report questionnaire. Eight domains of health are evaluated in the SF-36, each relating to a specific valence of health experience: bodily pain (BP), general health (GH), mental health (MH), physical functioning (PF), role limitation—emotional (RE), role limitation—physical (RP), social functioning (SF), and vitality (V). It has been used extensively in Australia for both population health and clinical applications and hence population norms exist for the SF-36. The Australian norms were collected in the 1995 National Health Survey as this instrument has not been used in subsequent national health surveys [21].

#### 3.4.2. Meditation Lifestyle Survey (MLS)

The Meditation Lifestyle Survey is an instrument developed specifically for this study. The aim of the survey was to provide a number of covariates to assist with the analysis of the SF36 data. The scales involved yes/no answers for demographic questions in addition to ordinal type questions about meditative and lifestyle practices. The factors were developed in consultation with a number of experienced meditators and experts to ensure face validity and interrater reliability.

Most of the items in the survey were aimed at assessing the frequency of meditative practice among meditation practitioners to determine their level of adherence to lifestyle factors associated with meditation. Potentially confounding variables in the practitioner's lifestyle were also included in the MLS Survey. The primary factor of interest was the experience of mental silence or “thoughtless awareness”. The rationale of the lifestyle practices associated with meditation is that they are intended to maximize the meditation practitioner's ability to tap into the mental silence experience. Conceptual validity analysis of the MLS (in the analysis and results section of this paper) demonstrates that it explained at least 75% of the variance in practitioners self-reported experience of mental silence, indicating that the MLS was effective in capturing the salient factors in the mediators' lifestyle.

At each meeting or retreat, participants who were prepared to participate in the study were asked to complete an SF-36 questionnaire and an MLS survey. Those who did not attend the meeting, but had been identified through informal lists, were contacted by a research assistant. If they had not previously completed a questionnaire and survey, they were mailed copies with a reply paid envelope. Each person was phoned by a research assistant (after four weeks) to maximize return rates.

### 3.5. Analysis

Statistical analysis was conducted using SPSS version 17.

To compare the scores on various subscales of the SF36 with the national normative data *t*-tests were conducted. We used a Holm-Bonferroni correction for multiple testing. The values presented in this report are those derived after this adjustment.

To explore the degree to which aspects of meditative practice contribute to health scores, the relationships between various demographic and meditative lifestyle factors from the MLS with outcomes in the SF-36 were examined using Pearson Product Moment correlations.

A multiple regression analysis was conducted to explore colinearity between the various demographic factors, meditative lifestyle factors, health scores, and the MS experience. The total SF-36 and certain subscales were selected as dependent variables. Since the MLS was constructed around the notion that the experience of mental silence is facilitated by the various practices and lifestyle factors featured as items in the MLS, these items were included in the regression analysis. In addition, demographic factors known to independently influence physical and mental health were also included as factors in the regression analysis. This included age, gender, relationship status, history of mental illness, educational level, ethnicity, consumption of alcohol, tobacco, marijuana, and other recreational drugs and social support. In this case frequencies of attending social gatherings and other meetings of meditators were interpreted as social support.

## 4. Results

### 4.1. Demographic Data

Demographics of the 343 meditation practitioners who participated in the study are detailed in Table 1. Meditation lifestyle data are summarised in Table 2.

### 4.2. The Experience of Mental Silence

Just over half of the sample (51.9%) claimed to experience the state of mental silence or “thoughtless awareness” for more than one or two minutes several times a day. A little over one-quarter (28.6%) experienced this state once or twice day. Approximately one-tenth, 11.3%, experienced mental silence once or twice per week. A much smaller proportion experienced it less often.

### 4.3. Functional Health

Functional health scores for the meditators were high. A total of 92% of the respondents described their health is as “good” or better (“excellent” (28.6%), “very good” (43.8%), or “good” (19%)). Nearly half, 48.6% described their health as “much better” or “somewhat better” than a year ago, while 45.7% described their health as “about the same” as a year ago, while 88.1% agreed that their health was “definitely” or “mostly” excellent.

**Figure 1:**
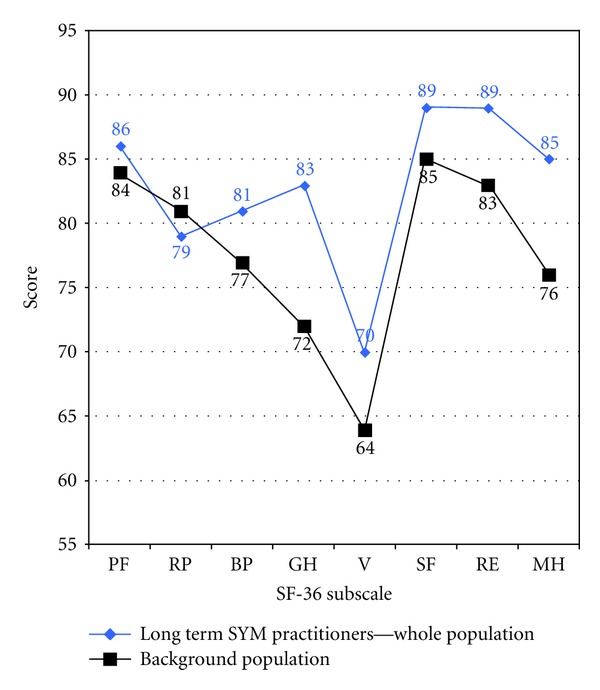
SF-36 polygon for Australian national norm data and mental silence sample.

A more precise understanding of the differences can be appreciated by inspecting the comparison of the Australian normative data for the SF-36 with the meditators' measures. These results are quite striking and are illustrated in Figure 1 and summarised in Table 3.

The largest differences appear to be in the mental health (MH), role-limitation emotional (RE), and general health (GH) subscales. All differences except physical functioning (PF) and role limitation physical (RP) were significant (*P* < 0.005) (see Table 3).

### 4.4. Correlations

**Figure 2:**
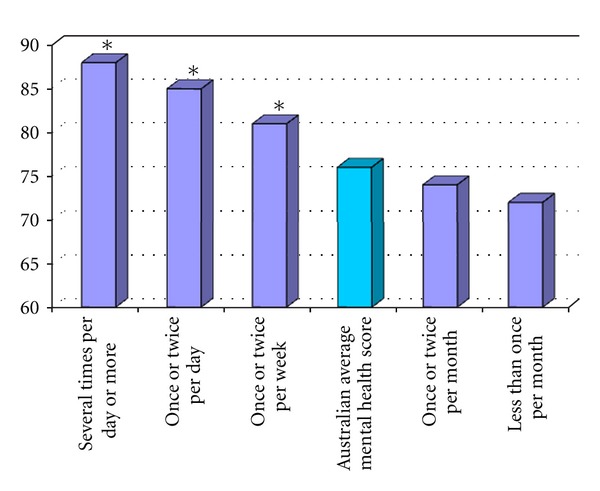
Mental Health subscale score for each category of frequency of mental silence (thoughtless awareness) experience.

To identify any potential relationships between the frequency of formal meditation (FM) and health, the means of the various SF-36 subscale scores were calculated for each FM category. No significant relationship between FM scores and SF-36 scores was observed. The means of the various SF-36 subscale scores were then calculated for each mental silence (MS) category to facilitate comparison. A linear relationship was apparent for the Mental Health (MH) subscale, which is illustrated graphically in Figure 2. A central notion in this study is the idea that the experience of mental silence may be a factor associated with the beneficial effects of meditation. In line with this, the simple correlations clearly demonstrate that MS is the factor most commonly and most strongly correlated with the various health scores. 

Pearson Product Moment calculations comparing the SF-36 total, the PCS, the mental health summary score (MCS), and the SF-36 subscales with MS raw scores were calculated revealing a number of notable correlations. These data are summarized in Table 4. 

Linear relationships with frequency of meditation were apparent for mental health (MH), general health (GH), vitality (V), mental health summary score (MCS), and SF-36 total score. The most clearly obvious linear association was with the MH subscale. 

Two demographic factors, salary and history of mental illness, were excluded from this investigation, because they were likely to be proxies for the dependent variables of interest. Several significant relationships became apparent. 

To develop an impression of the individual contribution of various constructs to the total variance in the SF-36 total score, a further set of GLMs was applied. The relationship between the MLS factors and the SF-36 total score (SF-36), the physical health summary score (PCS), the mental health summary score (MCS), and the eight subscales, was assessed. The strongest correlations occurred consistently in relation to mental silence. 

Comparing the MH score of each MS category demonstrates that the mean score of the first three categories for this sample is significantly higher than the national norm score for the same category (Table 5). 

### 4.5. Assessment of the MLS

One of the aims of this study was to investigate how the practice of meditation and, more specifically, the meditative experience of mental silence or “thoughtless awareness” may be related to mental and physical health outcomes. The experience of mental silence is thought to be facilitated by the various practices and lifestyle factors featured as items in the MLS. These include group meditation with other meditators, attending weekly meetings to socialize, meditate with, and learn more about meditation techniques, regular meditation, “foot soaking” (FS) and associated techniques, and duration of practice (DP). 

### 4.6. Conceptual Validity of the MLS

In order to evaluate the conceptual validity of this instrument, we tested the MLS and the relationship between the items (as independent variables) and MS (as the dependent variable). To explore the degree to which the MLS predicted MS when controlling for various demographic and MLS factors and their potential interactions, a general linear model (GLM) was used. In this model the independent variable was MS, and the dependent variables were duration of practice (DP), regular meditation, and foot soaking (FS). 

The covariates were all other MLS and demographic factors and a number of appropriately selected interactions between these covariates. The GLM had an *r* ^2^ of 0.736 and thus the model explained almost three-quarters of the variation. In this model, the significant factors were gender (*P* < 0.05), alcohol consumption (*P* < 0.005), marijuana/recreational drug consumption (*P* < 0.01), CM (*P* < 0.05), regular meditation (*P* < 0.05), duration of meditation practice (DP) (*P* < 0.005), the interaction between foot soaking (FS) and regular meditation (*P* < 0.005), and the interaction between FS and DP (*P* < 0.05). The *r* ^2^ value indicates that the MLS effectively captures the majority of factors that explain how often the practitioner experiences mental silence. 

To investigate the contribution of MS to the SF-36 score, a GLM was applied with only MS as the independent variable, resulting in an *r* ^2^ of 0.46. In order to contrast this with the individual contribution of FM, another GLM was applied with FM as the independent variable, resulting in an *r* ^2^ of 0.019. 

The relationships observed in this study are not entirely unprecedented. Meisenhelder's 2001 survey of Presbyterian ministers using the SF-36 as well as measures of prayer habits found that the sample had somewhat better health than national norms. Remarkably, frequency of prayer correlated significantly with the same subscales of the SF-36 in Meisenhelder's study as that for meditation in this study (Table 6). 

## 5. Discussion

The difference in scores on the SF-36 between the meditating population and the general Australian population is substantial and wide ranging. The apparent positive differences between the sample of meditation practitioners and the national norms may be the result of confounding factors. In an attempt to control for this, and although somewhat limited by the nature of the Australian National Health Survey dataset, even when comparing the health scores of that portion of the population that does not consume tobacco (the only factor that the ABS dataset allowed us to control for) but has the same age profile as the meditators sample, the significant differences persisted. 

George and colleagues noted in their discussion about the nature and quality of samples used in surveys and longitudinal studies of religious practices that more than half of the studies that address the relationship between religion and health are based on samples of older adults (60+ years of age) [22]. Epidemiological study of religiosity and its relationship to health is currently dominated by a Western, Judeo-Christian perspective. George further noted that these kinds of studies are usually conducted within limited geographic regions within the USA and are thus potentially influenced by regional variations in religious observance (e.g., Bible Belt states versus West Coast). In contrast, this study involved a national, representative sample of meditators, the sample group was relatively young (with a mean age of 37), and its outcomes were compared to national, census-based normative data. An additional strength of this study is that it examined non-Judeo-Christian religiosity in a country comparable to, although not geographically connected to, the USA, on a sample of respondents who are ethnically similar and yet religiously different. 

The meditator population may well be selected for those who are more motivated to achieve and maintain health. Various surveys have shown that people who use meditation and other forms of complementary and alternative medicine hold strong affiliations with holistic health philosophies and are highly motivated to seek out self-empowering health improvement strategies. It is quite possible that a population of long-term meditation practitioners would be highly selected for such people. Moreover, those practitioners who do not experience positive effects or even experience negative effects naturally desist from the practice and attrit from the meditating population, further improving the mean health scores of the remaining population. 

Surveys of this nature necessarily generate a level of expectancy among respondents. The responses of the long-term meditating population could have been influenced by the prospect of the survey results constituting a validation of their chosen lifestyle and belief system. Nevertheless, the fact that the overall pattern of response in the meditating sample follows a similar pattern to that of the Australian population provides some reassurance that this was not a major confounder. Furthermore, the data reported here are almost exactly the same as the data obtained in two pilot surveys. 

It is important to note that while modern science most commonly characterises meditation as a relaxation response or a pattern of specifically focused attention, these conceptualisations differ fundamentally from the authentic descriptions of the meditative experience originating in ancient India. The original source texts clearly state that a key defining feature of meditation is the experience of mental silence. For example, in what is probably the oldest known definition of meditation, the narrator explains in the ancient Indian Mahabharata that a meditator is “… like a log, he does not think” [23]. Similarly Lao Tse instructs the reader in the Tao Te Ching to “empty the mind of all thoughts”. Many other explicit examples of this idea can be found in Eastern literature from virtually every historical period. Yet Western definitions of meditation have consistently failed to acknowledge this crucial feature. 

The mechanisms by which meditation techniques exert their claimed effects are also unclear. One very popular view, which has become more or less the default explanation, is that the physiological changes are characterised by the relaxation response—that is, the physiological changes that occur during rest, characterised by reductions in heart rate, blood pressure, and respiratory rate and increases in skin temperature, skin resistance, and alpha wave activity in the brain are thought to be responsible. All of these are brought about by reducing activity of the sympathetic component of the autonomic nervous system and increasing activity of the parasympathetic components of the ANS. Psychophysiological studies of Sahaja Yoga, that is mental silence, however suggest that it does not elicit a typical relaxation response. For example, a study of skin temperature changes during a single Sahaja Yoga meditation session demonstrated a reduction in palmar skin temperature, which is the opposite of that predicted by the relaxation response model. A control group that was engaged in simple rest did manifest skin temperature increases. Yet there were no significant differences in heart rate between the two groups [24]. 

In her 2001 study, Meisenhelder proposed that the relationships observed between frequency of prayer and higher health scores could at least be partly caused by the relaxation effect of prayer and its consequent ability to ameliorate the effects of stress. This idea is supported by studies such as that by Carlson who studied the autonomic impact of Christian devotional meditation in an RCT design and found that it was as effective, and in some parameters more effective, as conventional relaxation [25]. 

So, it is noteworthy that both this study and the study by Meisenhelder and Chandler [1] report correlations in the same SF-36 subscales (general health, vitality, and mental health) suggesting that both prayer and meditation are both associated with similareffects. An interesting distinction however is that our study suggests that the experience of mental silence has a stronger (by a factor of approximately two to three) relationship with these dimensions as compared to the relationship that Meisenhelder reports between prayer and the same dimensions. The notion that this may be due to some inherent effect of the mental silence experience is supported by the two RCTs on work stress and asthma, mentioned earlier, and clearly warrants further investigation. 

The observed relationship between how often a meditator performed “formal meditation” and health measures was considerably weaker than for mental silence, implying that differences between contemplative practices (such as prayer or meditation) that are overtly similar but sometimes experientially distinct (i.e., mental silence versus mental activity) have significantly different health implications. 

The observed relationship between meditative practices and mental health is not as strong as for measures of physical health. In many ways, this might be expected since the intervention is primarily focused on a mental experience with the specific aim of reducing negative affect, thinking patterns, and related behaviours. Mood, thoughts, and behaviour patterns are in constant flux, much of it reflecting (and influencing) brain electrochemical activity and other neurobehavioural phenomena which change from moment to moment. 

There is evidence that meditation can have short- and long-term effects on both function and structural brain plasticity in addition to its already recognised ability to cause relaxation and reduce stress. Aftanas has shown that the practice of SYM, and the experience of mental silence, is strongly reflected in both brain electrophysiology and mood [26]. A study by the same group demonstrated reduced emotional reactivity in long-term meditators compared to controls which was reflected in psychological, physiological, and electrophysiological reactivity to standardised stressful stimuli presented in a video film. This provides evidence for the notion of “emotional detachment” and hence enhanced emotional stability and resilience to stressful events [27]. A smaller intervention study by Morgan over just 6 weeks showed a significant reduction in anxiety, depression, and related symptoms in patients with major depression compared to controls [17] which appears to reflect the clinical relevance of Aftanas's findings. This has broader implications particularly as understandings of the relationship between neuroplasticity and meditation emerge. Lazar studied a group of Buddhist meditators and found that meditators compared to controls had significantly increased cortical thickness in right middle and superior frontal cortex and insula suggesting that meditation is associated with delaying of the usually age-related thinning of right frontolimbic brain regions [28]. Hence it is quite possible that long-term meditation may facilitate both electrophysiological and structural changes in brain function that may explain why the population of long-term meditators that we studied manifested an apparent advantage as compared to the background population particularly in metal health scores. An excellent review and discussion paper by Rubia [29] discusses the possible neurobiological underpinnings of meditation and its potential role in mental health in detail that is not possible within the limits of this paper. 

These observations might also explain why mental health factors are much more likely to be immediately responsive to such an intervention whereas physical health factors, which rely significantly on anatomical structures and mechanical function, will take much longer to manifest (if at all) and are subject to a vast number of other environmental confounders that may obscure any such relationship. 

While we acknowledge that cross-sectional studies are prone to a number of confounders, the implications for population mental health are nevertheless worth considering. Given that neuropsychiatric disorders such as depression and substance abuse are increasing in incidence as well as their impact and that there are few long-term curative options for many of these conditions, there is merit in exploring the role of preventative strategies such as meditation. The findings of this study warrant further examination of meditative practices as a conceptually innovative preventative and therapeutic option for public mental health. The meditation technique assessed in this study is low/zero cost and to date has not been associated with any adverse effects; hence further exploration of this approach in enhancing general wellbeing, quality of life, and mental health would seem to be highly worthwhile. 

## 6. Conclusion

This is the first study to report a cross-sectional survey aimed at assessing health and quality of life in a population of people who meditate regularly and have done so for a long period of time. It is also the first study to explore the interrelationship between factors such as meditative experience, meditative practices, a “meditative” lifestyle, and health outcomes. 

Long-term Sahaja Yoga meditation practitioners appear to experience better quality of life and functional health than the general population. Perhaps most importantly is the observation that there appears to be a relatively robust and consistent relationship between the meditative experience of mental silence and health, especially mental health. Based on the premise and findings of this study, these observations necessarily apply to practitioners of mental silence-orientated forms of meditation of which Sahaja Yoga is an example. Taking into account the fact that two well-designed RCTs of mental silence also demonstrated significant effects on both mental and physical health parameters compared to active controls, the association between the subjectively reported experience of mental silence and health observed in this study is likely to be causal. Hence this survey data suggests that such approaches to meditation may have a potentially valuable role in primary mental health prevention. Further research to evaluate this possibility is clearly warranted. 

Future research involving comparison of mental-silence-orientated practitioners with other populations (controlling for the exclusion of health risk factors and similar lifestyle changes as well as religious and spiritual observances) would be useful to more clearly identify the source of the apparent benefits of that this population appears to enjoy.

## Figures and Tables

**Table 1:** Demographics of meditation practitioners.

Factor	Meditation practitioners*
Number of responses	343
% Male	39.6
Mean age and age range	44, (SD 13.4), range 18 yrs to 82 yrs
% Caucasian	77.2
% Asian	21.9
% Single/never married/divorced/widow	27
% Married/de facto	73
High school, highest level	25.9
Undergraduate, highest level	49.4
Postgraduate, highest level	21.2
No history of mental illness	87.9
History of minor mental illness	10.4
History of major mental illness	1.7

*In comparison, the National Health Survey in 1995 was weighted to population norms with the SF36 administered to only the household members aged 18 years or more. The mean age was 42.6 (SD 16.5) with 48% male.

**Table 2:** Meditation lifestyle of practitioners.

Meditation lifestyle data	Meditation practitioners
Total responses (*n*)	343
Mean duration of practice (years)	12.9
% who meditate regularly	95.6
% Formal meditation, twice/day	51.2
% Formal meditation, once/day	31.8
% Formal meditation, most days	12.1
% Formal meditation, once/week or less	5
% Group meditation, once/day or more	10.1
% Group meditation, most days	10.2
% Group meditation, once/week	60.5
% Socialize with other practitioners most days or more often	11.6
% Socialize with other practitioners once/week	37.8
% Socialize with other practitioners less than once/week	50.6
% who do not consume alcohol	92.3
% who do not smoke	92.6
% who do not use marijuana or other recreational drugs	98

**Table 3:** Comparison of SF-36 subscale scores between mental silence practitioners and Australian national norms.

Subscale	Test value (national norm)	*T*	Df	Significance	Mean difference
PF	83.82	1.75	345	0.082	2.39
RP	80.64	−0.95	345	0.342	−2.10
BP	77.05	3.19	345	0.002	3.79
GH	71.81	12.72	332	0.001	10.72
V	64.27	6.24	337	0.001	5.98
SF	85.25	3.59	337	0.001	3.52
RE	83.44	4.43	334	0.001	5.74
MH	75.75	14.26	336	0.001	9.56

**Table 4:** Correlation of frequency of mental silence experience (MS) and SF-36 scores.

		PF	RP	BP	GH	V	SF	RE	MH	PCS	MCS	Total SF-36
MS	Pearson Correlation	−0.039	−0.099	−0.005	−0.200**	−0.217**	−0.030	−0.097	−0.345**	−0.125*	−0.243**	−0.175**
	*n*	341	341	341	330	335	335	333	334	326	316	316

*n*: number of samples.

*: 0.05—Probability of a Type I error.

**: 0.01—Probability of a Type I error.

**Table 5:** Comparison of the MH score for each MS category with Australian normative values for MH score.

TA Category	Number in sample	MS category mean score	MS category score SD	*T*	Df	Significance	Mean difference
Several times per day	172	87.98	10.04	15.97	171	0.001	12.22
Once or twice per day	98	85.35	10.46	9.07	97	0.001	9.59
Once or twice per week	38	81.05	12.04	2.71	37	0.010	5.30
Once or twice per month	13	74.46	22.30	−0.21	12	0.838	−1.29
Less than once per month	13	71.69	21.45	−0.68	12	0.508	−4.06

**Table 6:** Comparison of SF-36 subscales study results with those of Meisenhelder and Chandler [1].

	Meisenhelder and Chandler [1]		This study	
Subscale	Frequency of prayer *r*	Significance	Frequency of mental silence *r*	Significance
PF	−0.001	0.965	−0.039	0.474
RP	−0.010	0.715	−0.099	0.067
BP	0.037	0.166	−0.005	0.928
**GH**	**0.088**	**0.001**	**0.200**	**0.001**
**V**	**0.103**	**0.001**	**0.217**	**0.001**
SF	0.027	0.317	−0.030	0.586
RE	0.039	0.154	−0.097	0.077
**MH**	**0.117**	**<0.001**	**0.345**	**0.001**
